# Study of early stage non-small-cell lung cancer using Orbitrap-based global serum metabolomics

**DOI:** 10.1007/s00432-017-2347-0

**Published:** 2017-02-06

**Authors:** Agnieszka Klupczynska, Paweł Dereziński, Timothy J. Garrett, Vanessa Y. Rubio, Wojciech Dyszkiewicz, Mariusz Kasprzyk, Zenon J. Kokot

**Affiliations:** 1 Department of Inorganic and Analytical Chemistry, Poznan University of Medical Sciences, Grunwaldzka 6 Street, 60-780 Poznan, Poland; 2 Department of Pathology, Immunology and Laboratory Medicine, College of Medicine, University of Florida, 1395 Center Drive, Gainesville, FL 32610 USA; 3 Department of Thoracic Surgery, Poznan University of Medical Sciences, Szamarzewskiego 62 Street, 60-569 Poznan, Poland

**Keywords:** Lung cancer, Global metabolomics, Metabolite profiling, Mass spectrometry, Orbitrap

## Abstract

**Purpose:**

The aim of the project was to apply ultra-high-performance liquid chromatography–quadrupole-Orbitrap-high-resolution mass spectrometry for serum metabolite profiling of non-small-cell lung cancer (NSCLC). This Orbitrap-based methodology has been applied for a study of NSCLC potential markers for the first time.

**Methods:**

After extraction using protein precipitation, sera were separated on the ACE Excel 2 C18-PFP (100 × 2.1 mm, 2.0 µm) column using gradient elution and analyzed within the range of 70–1000 *m*/*z*. Only patients with early stage disease (stages IA–IIB) were included in the study, providing opportunity to find biomarkers for early lung cancer detection. The resulting metabolite profiles were subjected to univariate and multivariate statistical tests.

**Results:**

36 features were found significantly changed between NSCLC group and controls after FDR adjustment and 19 were identified using various metabolite databases (in-house library, HMDB, mzCloud). The study revealed a number of NSCLC biomarker candidates which belong to such compound classes as acylcarnitines, organic acids, and amino acids. Multivariate ROC curve built using 12 identified metabolites was characterized by AUC = 0.836 (0.722–0.946). There were no significant differences in the serum metabolite profiles between two most common histological types of lung cancer—adenocarcinoma and squamous cell carcinoma.

**Conclusions:**

Through identification of novel potential tumor markers, Orbitrap-based global metabolic profiling is a useful strategy in cancer research. Our study can accelerate development of new diagnostic and therapeutic strategies in NSCLC. The metabolites involved in discrimination between NSCLC patients and the control subjects should be further explored using a targeted approach.

**Electronic supplementary material:**

The online version of this article (doi:10.1007/s00432-017-2347-0) contains supplementary material, which is available to authorized users.

## Introduction

Recently, the growing trend of metabolomic studies of cancer has been observed. It is well known that cancer cells exhibit an altered metabolism and increased energy requirements compared to normal cells. However, the tumor metabolome has not yet been fully explained (Aboud and Weiss 2013). In contrast to normal cells, which utilize oxidative phosphorylation for energy production, cancer cells gain energy from glycolysis occurring even in the presence of oxygen. This phenomenon is called the Warburg effect (Vander Heiden et al. 2009). Another distinctive trait of cancer cell metabolism is the use of glutamine as a main source of energy (Reitzer et al. 1979; DeBerardinis et al. 2008). The characteristic metabolic alterations occurring in tumors might result in changed levels of some metabolites in tissues and body fluids. Human biological fluids are commonly recognized as vehicles for the transmission of markers of many human disorders and thus the metabolite profiling of different body fluids is a promising strategy in cancer research. There is a multitude of potential applications of metabolomics in oncology, including early and accurate diagnosis, estimation of treatment efficacy, and development of novel anti-cancer therapies (Spratlin et al. 2009).

Application of high-throughput and sensitive analytical techniques that are used in metabolomics makes them powerful tools in the field of oncology and aids understanding what is happening in cancer cells. Given the complex nature of cancer, global metabolomic profiling offers the unique opportunity of broadening the knowledge on the tumor metabolome. In particular, there is an increasing interest in the study of metabolic changes related to lung cancer over the last few years (Hori et al. 2011; Chen et al. 2014; Deja et al. 2014; Puchades-Carrasco et al. 2016). Lung cancer constitutes one of the greatest challenges in contemporary oncology because of difficulties in early detection resulting in lung cancer as a leading cause of cancer death for many years (Subramaniam et al. 2013). Blood metabolite profiling of lung cancer includes the application of such analytical techniques as gas chromatography–mass spectrometry (Hori et al. 2011; Musharraf et al. 2015; Fahrmann et al. 2015), ultra-high-performance liquid chromatography–linear ion trap-mass spectrometry (Mazzone et al. 2016), high-performance liquid chromatography–quadrupole-time-of-flight-mass spectrometry (Chen et al. 2014; Li et al. 2014), and nuclear magnetic resonance (Deja et al. 2014; Puchades-Carrasco et al. 2016). In the above-mentioned studies, changes in many distinct groups of metabolites were reported, such as amino acids, carbohydrates, organic acids, fatty acids, and nucleotides. It should be noted that the results obtained in the previously published metabolomic research were not always in agreement with each other. One of the discrepancies exists regarding glutamate which was found elevated in blood of lung cancer patients by Fahrmann et al. (2015) and Puchdes-Carrasco et al. (2016), whereas Hori et al. (2011) reported a decreased level. Another discrepancy is in the lactic acid level. According to Hori et al. (2011) and Musharraf et al. (2015), higher level of lactic acid occurred in the plasma of patients with lung cancer, whereas Chen et al. (2014) observed its reduction in sera from lung cancer group as compared with controls. The possible reasons of the above-mentioned discrepancies can be associated with differences in analytical methodologies, sample handling, or clinical characteristics of patients. Therefore, it can be concluded that despite efforts made by many research groups, there are still several gaps and inconsistencies in the knowledge about the serum metabolome of lung cancer patients.

Only detection of early metabolic alterations can lead to early lung cancer diagnosis. Therefore, it is of particular interest to study the utility of cancer-related metabolic changes occurring at the beginning of the disease. Many of the metabolomic studies included late-stage cancer cases (Hori et al. 2011; Fahrmann et al. 2015; Miyamoto et al. 2015). Therefore, the putative biomarker metabolites identified in those studies would likely fail a further validation step for early detection of cancer. It should be noted that by including advanced stage patients in the research, the obtained results could be overoptimistic. Moreover, there is a risk, especially in the case of amino acids, that the observed abnormalities are influenced by nutritional deficiencies and accompanying weight loss experienced by many patients with advanced lung cancer (del Ferraro et al. 2012). The application of metabolomic research in the discovery phase for new cancer biomarkers is indisputable; however, the selection of representative samples has a major impact on any conclusions drawn from the research.

The aim of the study was to apply an Orbitrap-based global metabolomic approach to the analysis of serum of patients with non-small-cell lung cancer (NSCLC). To our knowledge, this is the first study which presents the application of ultra-high-performance liquid chromatography–quadrupole-Orbitrap-high-resolution mass spectrometry (UHPLC-Q-Orbitrap-HRMS) for searching NSCLC potential markers. Until now, Orbitrap-based profiling has only been used to compare two metabolomes (plasma and serum) of small-cell lung cancer (SCLC) patients undergoing treatment with standard chemotherapy (Wedge et al. 2011) and to characterize KRAS mutants in NSCLC cells (Brunelli et al. 2014). The current study provided new data on metabolites, which could translate to the improvement of lung cancer diagnosis and treatment. The study involved serum analysis of patients with newly diagnosed NSCLC prior to initiation of a therapy and a matched control group. Only patients with early stage disease (stage IA–IIB) were included to the study, representing an opportunity to define biomarkers for early lung cancer detection.

## Methodology

### Reagents and materials

LC/MS grade acetonitrile, 0.1% formic acid in water, methanol, and acetone were purchased from Fischer Scientific Co. (Pittsburgh, PA, USA). Isotopically labeled standards: creatine-D3, l-leucine-D10, l-tryptophan-2,3,3-D3, and caffeine-D3 were obtained from CDN Isotopes Inc. (Pointe-Claire, Quebec, Canada). Mixture of amino acids was purchased from Sigma-Aldrich (St. Louis, MO, USA).

### Study population

The project was approved by the ethics committee at the Poznan University of Medical Sciences (Decision No. 200/13). All participants provided a written informed consent for inclusion to the study. Fifty lung cancer patients were recruited in the Department of Thoracic Surgery, Poznan University of Medical Sciences. NSCLC diagnosis was conducted by the histopathological examination of tissue samples. Patients with other tumors were excluded from the study. The cancer staging was performed according to the seventh edition of the TNM staging system [tumor size, node involvement, and metastasis presence (Goldstraw et al. 2007)]. No anti-cancer treatment had been applied to the enrolled subjects. Twenty-five individuals included in the control group were recruited from subjects without cancer and chronic metabolic diseases who underwent a routine periodic medical examination. Data regarding demographic and clinical characteristics of study participants are presented in Table 1.

**Table 1:** Characteristics of the study participants

Parameter	Non-small-cell lung cancer	Control group
*N*		
Total	50	25
Male	28 (56.0%)	14 (56.0%)
Female	22 (44.0%)	11 (44.0%)
Age, year		
Mean	65	64
Range	53–86	50–78
BMI, kg/m^2^		
Mean	26.5	25.8
Range	17.6–33.9	21.1–34.6
Histological type		
Adenocarcinoma	25	–
Squamous cell	25	–
Stage^a^		
IA	17	–
IB	25	–
IIA	6	–
IIB	2	–
Tumor grade		
G1	2	–
G2	27	–
G2/G3	2	–
G3	15	–
Unknown	4	–

^a^According to TNM classification (Goldstraw et al. 2007)

### Sample collection and preparation

The sera were collected in the same manner from both groups of subjects (cancer patients and controls) in the morning following an overnight fast using S-Monovette tubes (Sarstedt, Nümbrecht, Germany) with a clotting activator according to the manufacturer’s instruction. The obtained sera were aliquoted and stored at −80 °C until use. For sample preparation, the following protein precipitation procedure was used. 100 μL of thawed sample was mixed with 20 μL of isotopically labeled internal standard solution. Then, 800 μL of acetonitrile:methanol:acetone (8:1:1, v/v/v) solution was added to precipitate proteins, followed by incubation at 4 °C for 30 min to further precipitate proteins. Next, the samples were centrifuged at 20,000 RCF for 10 min at 8 °C and 750 μL of the supernatant was transferred to a new microcentrifuge tube. The supernatant was dried under a gentle nitrogen flow using MULTIVAP nitrogen evaporator (Organomation Associates, Inc., Berlin, MA, USA) and then reconstituted by adding 100 μL of 0.1% formic acid in water followed by incubation on an ice bath for 15 min. The sample was then centrifuged at the same conditions, and the supernatant was transferred to a vial. To avoid possible bias and batch effect, all samples were prepared in random order on the same day as one batch. Samples were randomized also for the following UHPLC-Q-Orbitrap analyses. Aliquots of sample extracts representing different groups were mixed to obtain pooled samples. They were used as quality control (QC) samples as well as to acquire MS/MS spectra.

### UHPLC-Q-Orbitrap-HRMS analysis

Global metabolite profiling was performed using a high-resolution, accurate-mass Q Exactive Hybrid Quadrupole-Orbitrap mass spectrometer (Thermo Fisher Scientific, San Jose, CA, USA) equipped with a heated electrospray ion source coupled to a UHPLC UltiMate 3000 (Dionex Corporation, Sunnyvale, CA, USA). The chromatographic separation was conducted using the ACE Excel 2 C18-PFP (100 × 2.1 mm, 2.0 µm) column (Advanced Chromatography Technologies, Aberdeen, Scotland) and a gradient elution. The method used 0.1% formic acid in water and acetonitrile as eluents A and B, respectively. The gradient program was 0–3 min 100% A, 3–13 min linear from 100 to 20% A, 13–16 min 20% A, 16–16.5 min linear from 20 to 100% A, and 16.5–20.5 min 100% A. The flow rate was initially maintained at 0.35 mL/min, at 16.8 min increased to 0.6 mL/min, and at 20 min decreased back to 0.35 mL/min. The autosampler temperature was maintained at 4 °C and the column at 25 °C. Injection volume was 2 μL. The mass spectrometer was operating in full MS mode at a mass range of 70–1000 *m*/*z* in positive ionization. In addition, several pooled samples were analyzed in data-dependent MS/MS mode to obtain MS/MS spectra of the most intensive signals (top 5). Other mass spectrometry parameters were summarized in Table 2.

**Table 2:** Selected parameters of the Q-Orbitrap system used for the analysis of serum samples

Parameter	Setting
Probe temperature	350 °C
Spray voltage	3500 V
Capillary temperature	320 °C
Sheath gas flow	40 arb
Auxiliary gas flow	10 arb
Sweep gas flow	1 arb
Mass resolution	35,000 at *m*/*z* 200
Source collision-induced dissociation	2 eV

### QC assessment

Two types of QC samples were used to assure the quality and reliability of acquired data. Two pooled QC samples (representing NSCLC and controls) were injected five times throughout the course of the sequence of analyses. Neat QC sample consisting of a mixture of several labeled internal standards, and unlabeled metabolites were injected ten times throughout the course of the sequence.

### Data processing and analysis

The obtained raw LC-HRMS data files were converted to mzML files using MSConvert from ProteoWizard (Chambers et al. 2012). The converted files were processed using the MZmine 2.19 software (Pluskal et al. 2010). Data processing comprised of several steps, i.e., mass detection, chromatogram builder, smoothing, chromatogram deconvolution, grouping of isotopic peaks, peak alignment with *m*/*z* tolerance of 10 ppm and retention time tolerance of 0.2 min, gap filling to fill in missing peaks, duplicate peak removing, and peak filtering (retention time range 0.7–17.0 min, peak duration range 0.0–2.0 min). The resulting peak list contained multiple missing values as well as gap-filled values in addition to detected peaks. Thus, the peak list was filtered to keep only those features that were detected in at least 90% of samples, which yielded a peak list containing 233 features.

The resulting metabolite profiles were subject to univariate and multivariate statistical tests in MetaboAnalyst 3.0 web server (http://www.metaboanalyst.ca) (Xia et al. 2015). Before multivariate (principal component analysis, PCA) and univariate (*t* test) analyses, data were filtered based on interquantile range that resulted in removing 5% of the variables that were unlikely to be of use when further modeling the data (variables of very small or near-constant values throughout the sequence). Data sets were then normalized by sum, log-transformed, and auto scaled. Fold change was calculated before columnwise normalization was performed. In the case of *t* tests, multiple hypotheses testing correction was performed by controlling the false discovery rate (FDR). The significance threshold for FDR was set to 0.05. Before univariate ROC curve analyses that utilized only data of the selected metabolites, columnwise data normalization was not performed. Multivariate ROC curves were generated by Monte Carlo cross validation.

To identify selected metabolites, several databases were used, i.e., the in-house library of standards analyzed previously using the same instrument and the same method, Human Metabolome Database (HMDB, http://www.hmdb.ca/), and advanced mass spectral database—mzCloud (https://www.mzcloud.org/). Using the in-house library, compounds were identified based on *m*/*z* and retention time (with *m*/*z* tolerance of 10 ppm and retention time tolerance of 0.2 min). In the case of HMDB, *m*/*z* was used to perform putative identification. Acquired MS/MS spectra and precursor m/z were used for identification in mzCloud.

## Results

### Characteristics of the study participants

The study group consisted of patients with NSCLC in the age range from 48 to 86. Based on histopathological findings, the research involved 25 lung adenocarcinomas and 25 lung squamous cell carcinomas. Patients with stage I lung cancer consisted of 84.0% of the study group. None of the patients had stage III or IV cancer. All subjects were Caucasians of Polish origin. The control group corresponded to the NSCLC patients in terms of sex, age, BMI, and ethnic origin. There were no significant differences in the samples analyzed in age (*p* = 0.5514) and BMI (*p* = 0.5959). Both groups had identical percentage of men and women. The characteristics of the cancer patients and control individuals are summarized in Table 1.

### QC samples

QC assessment is an important part of any experiment. In case of LC-HRMS data, variations between samples may come from the sample preparation step and variations between injections and/or the order of acquisition (sensitivity changes and carry over). To ensure the quality of data acquisition and to monitor the performance of the instrument, sample preparation and acquisition were randomized, internal standards were added to the samples, and pooled QCs, and neat QC were used. Analysis of internal standards and QC samples confirmed that the instrument drift was minimal. Pooled QCs were clustered on the PCA score plot (Online Resource 1). Thus, the acquired data could be used for further data analysis.

### Identification of differences in serum metabolite profiles between NSCLC patients and control subjects

Initial PCA analysis revealed that two samples representing two NSCLC patients were outliers (Online Resource 1). Those samples were subsequently removed from data matrix to prevent them from hampering the results of statistical analyses.

**Fig. 1:**
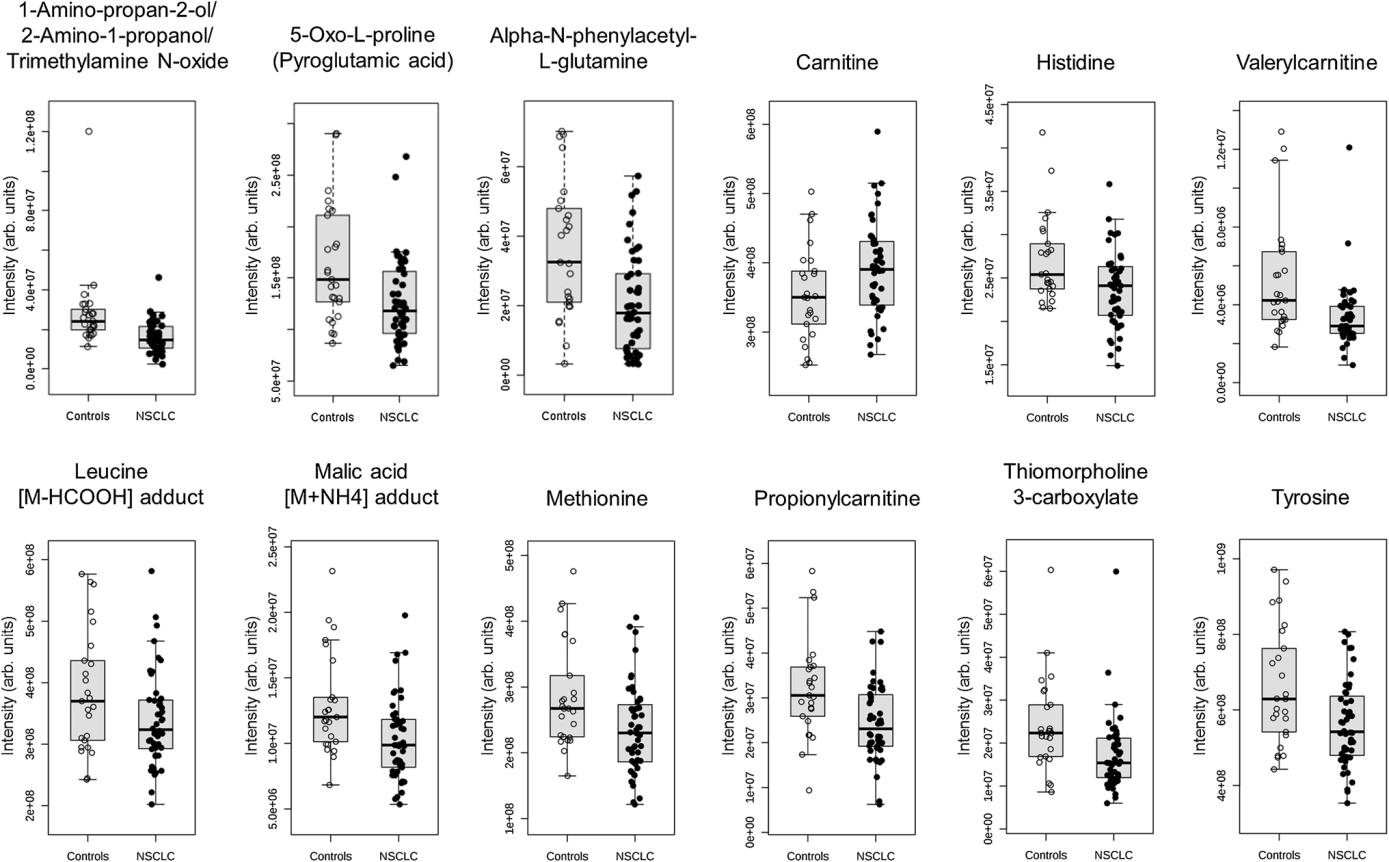
*Boxplots* of the identified metabolites. *NSCLC* non-small-cell lung cancer

78 features had *p* values from the *t* test below 0.05. The correction for multiple hypotheses testing revealed that among those features, 36 had FDR values below the threshold when the NSCLC group was compared with the control group (Table 3). An attempt was made to identify those features using various metabolite databases (in-house library, HMDB, mzCloud). As a result, 19 out of 36 features were identified either as a molecular ion of a metabolite or as an adduct of a metabolite (Table 3). In several cases, more than one entity of a particular metabolite has been identified. Thus, overall, 12 unique metabolites were identified: four amino acids (histidine, leucine, methionine, and tyrosine), two organic acids (pyroglutamic acid and malic acid), carnitine and two acylcarnitines (valerylcarnitine and propionylcarnitine), alpha-*N*-phenylacetyl-l-glutamine, thiomorpholine 3-carboxylate, and 1-amino-propan-2-ol/trimethylamine *N*-oxide/2-amino-1-propanol. In case of the last feature, it was not possible to indicate which of the three isomers had the highest probability of correct identification. Figure 1 presents boxplots of the identified metabolites, levels of which differed significantly between the NSCLC patients and the control group. Mean serum level of carnitine in the NSCLC patients was higher than in healthy controls, while the rest of the identified metabolites had mean levels lower in the NSCLC group as compared to the control group (Table 3; Fig. 1).

**Table 3:** List of metabolites had FDR values below the threshold (0.05)

Metabolite	RT	*p* value^a^	FDR	Fold change^b^	Identification
Identified metabolites					
Histidine	0.71	0.00127	0.01002	0.90	In-house library
1-Amino-propan-2-ol/2-Amino-1-propanol/Trimethylamine *N*-oxide	0.85	0.00004	0.00087	0.57	HMDB (1-Amino-propan-2-ol/Trimethylamine N-oxide), mzCloud (2-Amino-1-propanol)
Carnitine	0.88	0.00249	0.01772	1.12	In-house library
Thiomorpholine 3-carboxylate	1.25	0.00045	0.00553	0.72	HMDB
Malic acid: [M + NH_4_] adduct	1.33	0.00049	0.00565	0.81	HMDB
Methionine: [M–NH_3_] adduct	1.33	0.00079	0.00794	0.82	In-house library
Methionine	1.33	0.00121	0.01002	0.83	In-house library
Methionine: [M–HCOOH] adduct	1.33	0.00427	0.02859	0.84	In-house library
5-Oxo-l-proline (pyroglutamic acid)	1.79	0.00035	0.00454	0.79	In-house library
5-Oxo-l-proline (pyroglutamic acid): [M + Na] adduct	1.80	0.00126	0.01002	0.78	In-house library, HMDB
Leucine: [M–HCOOH] adduct	2.30	0.00612	0.03867	0.90	In-house library
Tyrosine	3.16	0.00007	0.00130	0.87	In-house library
Tyrosine: [M–NH_3_] adduct	3.16	0.00022	0.00318	0.88	In-house library
Tyrosine: [M–NH_3_–HCOOH] adduct	3.16	0.00063	0.00668	0.85	In-house library
Tyrosine: [M + Na] adduct	3.16	0.00126	0.01002	0.78	In-house library, HMDB
Tyrosine: [M–NH_3_–H_2_O] adduct	3.16	0.00266	0.01838	0.89	In-house library
Propionylcarnitine	6.50	0.00221	0.01652	0.77	In-house library
Valerylcarnitine	7.91	0.00005	0.00103	0.66	In-house library
Alpha-*N*-phenylacetyl-l-glutamine	8.00	0.00110	0.01002	0.58	HMDB, mzCloud
Unidentified metabolites					
*m*/*z* 193.0021	1.18	0.00457	0.02970	1.11	
*m*/*z* 140.9874	1.20	0.00026	0.00358	0.66	
*m*/*z* 162.0584	2.72	0.00103	0.00993	0.65	
*m*/*z* 123.0441	3.16	0.00010	0.00164	0.88	
*m*/*z* 157.0841	5.95	<0.00001	<0.00001	0.28	
*m*/*z* 195.1215	6.60	<0.00001	<0.00001	1.79	
*m*/*z* 217.1051	6.60	<0.00001	<0.00001	1.99	
*m*/*z* 157.0837	6.76	0.00063	0.00668	1.33	
*m*/*z* 239.1493	7.04	<0.00001	<0.00001	2.00	
*m*/*z* 261.1310	7.04	<0.00001	<0.00001	1.96	
*m*/*z* 256.1755	7.04	<0.00001	<0.00001	2.07	
*m*/*z* 300.2013	7.31	<0.00001	<0.00001	2.26	
*m*/*z* 344.2281	7.52	<0.00001	<0.00001	2.09	
*m*/*z* 180.1597	8.76	0.00015	0.00241	0.84	
*m*/*z* 163.1330	8.76	0.00224	0.01652	0.88	
*m*/*z* 239.0900	8.86	0.00701	0.04303	0.61	
*m*/*z* 282.2787	16.13	<0.00001	<0.00001	14.99	

The analyzed groups were patients diagnosed with early NSCLC and healthy controls

*RT* retention time; *FDR* false discovery rate

^a^Comparison type: NSCLC/controls

^b^According to *t* test

**Fig. 2:**
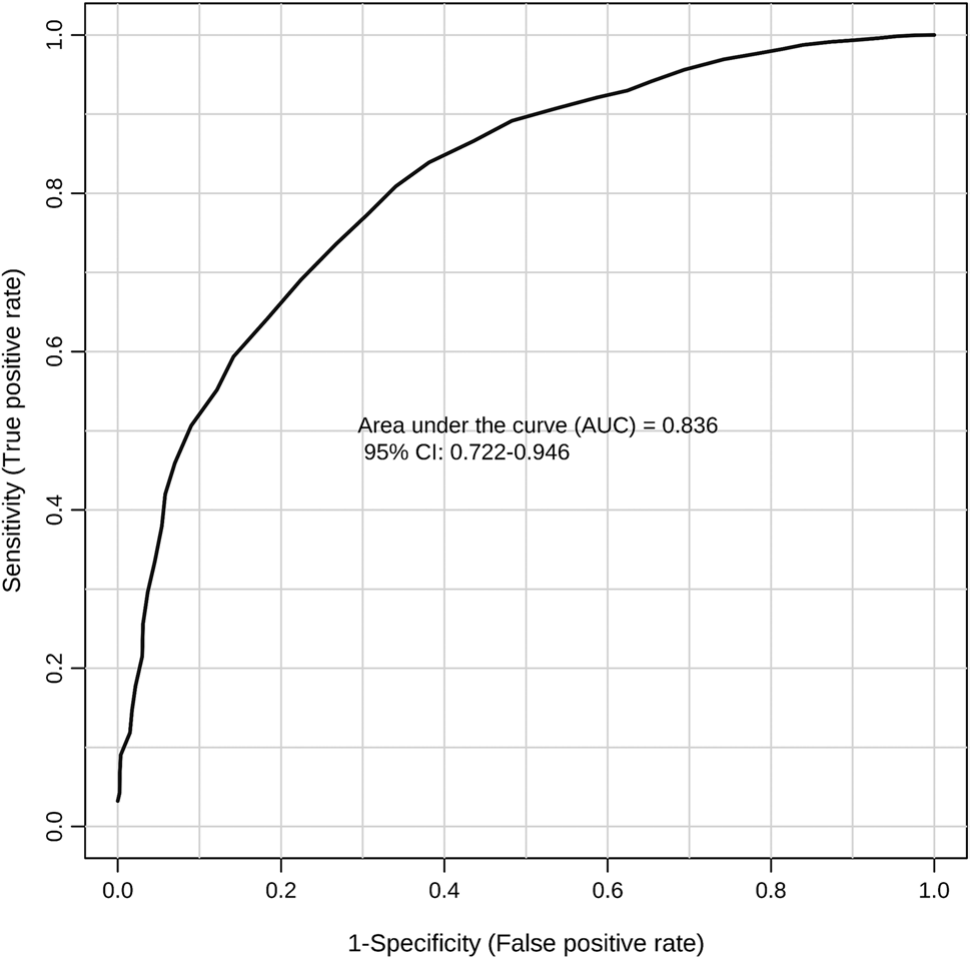
Multivariate ROC curve built using 12 identified metabolites. The analyzed groups were patients diagnosed with early non-small-cell lung cancer and healthy controls

Univariate ROC curve analyses were performed using data of the identified metabolites only. Table 4 shows the obtained areas under the curve (AUCs). The highest AUC was obtained for 1-amino-propan-2-ol/trimethylamine *N*-oxide/2-amino-1-propanol (0.799). The compound with the highest sensitivity was thiomorpholine 3-carboxylate (0.75), whereas the highest specificity was obtained for 1-amino-propan-2-ol/trimethylamine *N*-oxide/2-amino-1-propanol and carnitine (0.76) (Table 4). In addition, fold change and FDR values for that metabolite suggested that these differences were the biggest and the most statistically significant amongst all the identified compounds. Multivariate ROC curve analysis was also performed using the same data set of the 12 compounds. AUC for the obtained multivariate model was 0.836 (Fig. 2). Thus, the classification of the samples using the set of 12 metabolites was more effective than using a single metabolite.

**Table 4:** Area under the curve (AUC) values obtained in univariate ROC curve analyses of the identified metabolites (confidence intervals are shown in brackets) along with their sensitivity and specificity values

Metabolite	AUC	Sensitivity	Specificity
1-Amino-propan-2-ol/Trimethylamine *N*-oxide/2-Amino-1-propanol	0.799 (0.686–0.892)	0.71	0.76
5-Oxo-l-proline (pyroglutamic acid)	0.705 (0.560–0.813)	0.63	0.72
Alpha-*N*-Phenylacetyl-l-glutamine	0.731 (0.608–0.835)	0.56	0.80
Carnitine	0.656 (0.511–0.776)	0.52	0.76
Histidine	0.687 (0.549–0.813)	0.56	0.68
Valerylcarnitine	0.754 (0.616–0.865)	0.71	0.68
Leucine: [M–HCOOH] adduct	0.628 (0.494–0.765)	0.65	0.60
Malic acid: [M + NH_4_] adduct	0.717 (0.599–0.843)	0.71	0.64
Methionine	0.685 (0.541–0.795)	0.60	0.68
Propionylcarnitine	0.727 (0.598–0.837)	0.67	0.72
Thiomorpholine 3-carboxylate	0.734 (0.588–0.853)	0.75	0.68
Tyrosine	0.681 (0.545–0.799)	0.58	0.72

The analyzed groups were patients diagnosed with early NSCLC and healthy controls

In addition, the data set was reanalyzed consisting of NSCLC patients only. The aim was to compare the two types of NSCLC: adenocarcinoma and squamous cell carcinoma. None of the features had an FDR value below the threshold showing no significant difference between adenocarcinoma and squamous cell carcinoma.

## Discussion

In the current study, an Orbitrap-based metabolomic analysis of serum was performed to investigate whether useful data could be obtained to detect NSCLC and to understand underlying mechanisms. The research focused on the early changes in the serum metabolome caused by the development of lung tumor. Our findings indicated that patients with early stage NSCLC (stage IA–IIB) exhibited different serum levels of such metabolite classes as amino acids and their derivatives, acylcarnitines, and organic acids as compared with a healthy control group (Table 3).

As can be seen in Fig. 2, a set of 12 identified metabolites showed a promising ability to classify patients with NSCLC. Their combined AUC was equal to 0.836, which proves the usefulness of the proposed panel in detecting NSCLC. It can be supposed that the identification of the rest of significant features in the obtained metabolic profile will increase the discriminating potential of the multi-metabolite panel that we currently proposed. We did not include any of the unidentified compounds in the multivariate classification model, since without identification, it is not known whether they are endogenous metabolites or some artifacts of a different origin. Moreover, it is possible that the unidentified features do not correspond to unique compounds and more entities of particular compounds are present (i.e., molecular ions, adducts, and in-source fragments). Including such features to the model could lead to duplication of information and redundancy in data set.

One of compound classes, which was found to correlate with an NSCLC presence, is carnitine and its acylesters. The main function of acylcarnitines is fatty acid transport into the mitochondria for cellular energy production via β-oxidation (Kimhofer et al. 2015). As acylcarnitines can pass through mitochondrial and cell membranes, they are easily detectable in human blood (Millington and Stevens 2011). In our study, elevated level of carnitine and reduced levels of valerylcarnitine and propionylcarnitine were indicated as blood metabolic signatures of NSCLC. Recently, urine metabolic profiling of NSCLC patients revealed significant alterations of carnitine and 11 acylcarnitines (Wu et al. 2014). In that study of urine metabolome, carnitine was upregulated, while some medium-chain acylcarnitines were downregulated in cancer patients when compared to the control group. The abnormalities in the group of acylcarnitines were also observed in urine of patients with kidney and hepatocellular cancer (Kimhofer et al. 2015; Ganti et al. 2012). Experiments performed on a mouse xenograft model of human kidney cancer showed that acylcarnitnes are characterized by cytotoxicity and immune modulatory properties which are favorable for tumor growth and survival (Ganti et al. 2012). The role of acylcarnitines in NSCLC requires in-depth investigation; however, our study demonstrated the value of this group of metabolites in lung cancer biomarker studies.

Among the potential biomarker metabolites, which occurred at significantly different levels in serum of NSCLC patients compared to the control subjects, there are two low-molecular-weight organic acids: malic acid and pyroglutamic acid (also known as 5-oxoproline). Alterations in organic acid profiles in blood of lung cancer subjects were also found in some previous metabolomic studies; however, they were usually related to elevated lactic acid (Hori et al. 2011; Puchades-Carrasco et al. 2016; Musharraf et al. 2015). The current research demonstrated a high potential of pyroglutamic acid as an early marker of NSCLC. This acid is an intermediate metabolite of the gamma-glutamyl cycle of glutathione (l-*γ*-glutamyl-l-cysteinyl-glycine; GSH) production (Balendiran et al. 2004; Geenen et al. 2011). GSH is supposed to play many roles in regulating cancer development and growth, such as cell proliferation, apoptosis, and resistance to antineoplastic drugs. Therefore, GSH-related enzymes have attracted the attention of scientists as promising targets for medical intervention (Balendiran et al. 2004; Traverso et al. 2013; Ortega et al. 2011; Estrela et al. 2006). Malic acid is an intermediate of the Krebs cycle, which is a critical step in energy production by cells. Increased serum level of malic acid was reported in lung cancer patients by Hori et al. (2011). They also showed that the disease progression is characterized by a further increase in the concentration of malic acid. Due to the fact that our study only included subjects with early stage cancer, the findings of Hori et al. (2011) cannot be fully verified. Therefore, further targeted metabolomic investigation involving determination of low-molecular-weight organic acids is needed. To date, only one article presenting quantitative analysis of serum concentrations of six selected organic acids in NSCLC has been published; however, the analysis did not contain malic acid (Klupczynska et al. 2016b).

The amino acid that deserves special attention as a cancer biomarker candidate is histidine, which was found downregulated in serum of the NSCLC group. The significantly decreased level of histidine observed in the blood of lung cancer patients was also observed by Puchades-Carrasco et al. (2016) and Miyamoto et al. (2015), and may result from its excessive degradation or decreased synthesis. Histidine is converted to histamine in a reaction catalyzed by histidine decarboxylase (HDC). Thus, HDC constitutes a key regulator of histamine concentration. The biogenic amine histamine is involved in many physiological and pathological responses, including gastric acid secretion, inflammation, allergic reaction, and angiogenesis (Medina et al. 2003; Ghosh et al. 2002). Moreover, histamine is thought to be involved in inhibition of the local immune response against cancer (Masini et al. 2005). Although the hypothesis concerning the role of histamine in carcinogenesis was proposed in the 1960s, the mechanisms by which histamine and HDC are involved in cancer progression remain poorly understood. Both HDC and histamine content were found significantly higher in the colorectal cancer specimens when compared to normal mucosa (Masini et al. 2005). The elevated expression of HDC was also identified in human melanoma (Haak-Frendscho et al. 2000) and small-cell lung carcinoma (Matsuki et al. 2003; Graff et al. 2002). Our study provides evidence that the cancer metabolome is reflected in body fluids and measuring blood histidine concentration, a precursor for histamine, can provide a valuable information due to its association with the presence of cancer. It is also noteworthy that the significantly decreased blood concentration of histidine was observed in other cancer types, such as gastric cancer, colorectal cancer, and breast cancer (Miyagi et al. 2011). Therefore, it can be supposed that a decline in histidine blood level is a characteristic feature common to many types of cancer, not only NSCLC.

Our results confirmed the occurrence of metabolite alterations is serum of lung cancer subjects. Moreover, we indicated that some abnormalities in the metabolic profile of NSCLC patients are apparent at the beginning of the disease. The class of metabolites, which is currently the most explored in blood of NSCLC patients, is free amino acids. Some targeted metabolomic profiling focusing on determination of free amino acid levels in NSCLC has appeared in recent years (Klupczynska et al. 2016a; Kim et al. 2015; Shingyoji et al. 2013; Maeda et al. 2010). The analyses of amino acid profiles did not reveal one specific molecule that can play a role of NSCLC marker, but they emphasized the potential of using a multi-marker panel in lung cancer detection. Based on our study, it can be concluded that further study of the alterations in organic acid and acylcarnitine blood profiles in NSCLC is needed, since the metabolites from these two groups can represent valuable components of a multi-metabolite classifier useful in NSCLC diagnosis.

Finally, our research demonstrated that there are no significant differences in the serum metabolite profile between patients with lung adenocarcinoma and patients with squamous cell lung carcinoma. It is still unclear whether each histological type of lung cancer has its own distinct metabolic profile, because data available in the literature are inconsistent. Hori et al. (2011) reported differences in the serum metabolic profile of patients with various types of lung cancer; however, they did not apply multiple hypothesis testing correction. In contrast, Mazzone et al. (2016) used adjustment for multiple comparisons and observed no significant variations in serum metabolite levels between these two main histological types of lung cancer. To avoid false positives, we also applied multiple comparison approach in the study. Our work supports the hypothesis that serum metabolic profile is independent to NSCLC histological type.

It can be concluded that through identification and characterization of novel potential tumor markers, Orbitrap-based global metabolic profiling is a useful strategy in cancer research. Our research revealed a number of significant changes in serum of NSCLC patients with early stage of the disease, which can accelerate development of new diagnostic and therapeutic strategies. However, each study has its own strengths and limitations. One of the strengths of our research is the application of Orbitrap mass analyzer for the first time in NSCLC serum profiling. Moreover, we used FDR adjustment for data analysis that controls for the overall probability of a type I error. Adjustment for multiple comparisons is a particularly relevant issue in untargeted metabolomics, where hundreds of univariate tests are performed; therefore, the selection of biomarker candidates based on their FDRs, not on raw p values, is recommended. The study was restricted to patients with stage I and II cancer to reduce the influence of late-stage cancer cases on the obtained metabolite profiles. In addition, we examined two most common histological types of lung cancer and the presented findings apply to both types, as it was proven by statistical analyses. The results of our study are promising; however, only 19 out of 36 significantly altered low-molecular-weight molecules were identified. Another weakness of this research is a lack of validation of our results using a separate set of samples. As a part of the biomarker discovery phase, this study requires verification in larger patient cohorts from various institutions to estimate the robustness of the observed metabolite abnormalities. It should also be noted that the presented data are acquired through the semi-quantitative global metabolomic analysis, thus to better estimate the magnitude of variances in the selected metabolite levels, the application of a targeted metabolomic research, providing their absolute concentrations, is needed. Addressing the above-mentioned limitations is an aim for future research.

## Conclusions

When compared with previous studies, our research was focused on investigation of metabolic changes accompanying NSCLC at early stage of the disease (stage I and stage II). The presented analytical methodology has been used for the analysis of NSCLC for the first time and revealed a number of biomarker metabolites which belong to such classes as acylcarnitines, organic acids, and amino acids. The metabolites involved in discrimination between NSCLC patients and the control subjects should be further explored using targeted approach with the application of triple quadrupole mass spectrometry to prove their clinical usefulness. Another future direction of research should be related to a deeper elucidation of the identified metabolites in NSCLC biology. The advances in the molecular understanding of the roles of the particular metabolites in the neoplastic process could lead to development of novel therapeutic tools against NSCLC.

## Electronic supplementary material

Below is the link to the electronic supplementary material.

Supplementary material 1 (PDF 188 KB)
