# Supplementary material for: Study of early stage non-small-cell lung cancer using Orbitrap-based global serum metabolomics
Source: J Cancer Res Clin Oncol. 2017 Feb 6;143(4):649–59. doi: 10.1007/s00432-017-2347-0 (PMC5352735; doi:10.1007/s00432-017-2347-0)
Supplement: Supplementary file 1 — Supplementary material 1 (PDF 188 KB) [file 432_2017_2347_MOESM1_ESM.pdf]

Supplementary Material

Study of early stage non-small cell lung cancer using Orbitrap-based global serum metabolomics

Journal of Cancer Research and Clinical Oncology

Agnieszka Klupczynska, Paweł Dereziński, Timothy J. Garrett, Vanessa Y. Rubio, Wojciech Dyszkiewicz, Mariusz Kasprzyk, Zenon J. Kokot

Corresponding author: Prof. Zenon J. Kokot, Ph.D., Department of Inorganic and Analytical Chemistry, Poznan University of Medical Sciences, Grunwaldzka 6 Street, 60-780 Poznan, Poland, email address: zkokot@ump.edu.pl.

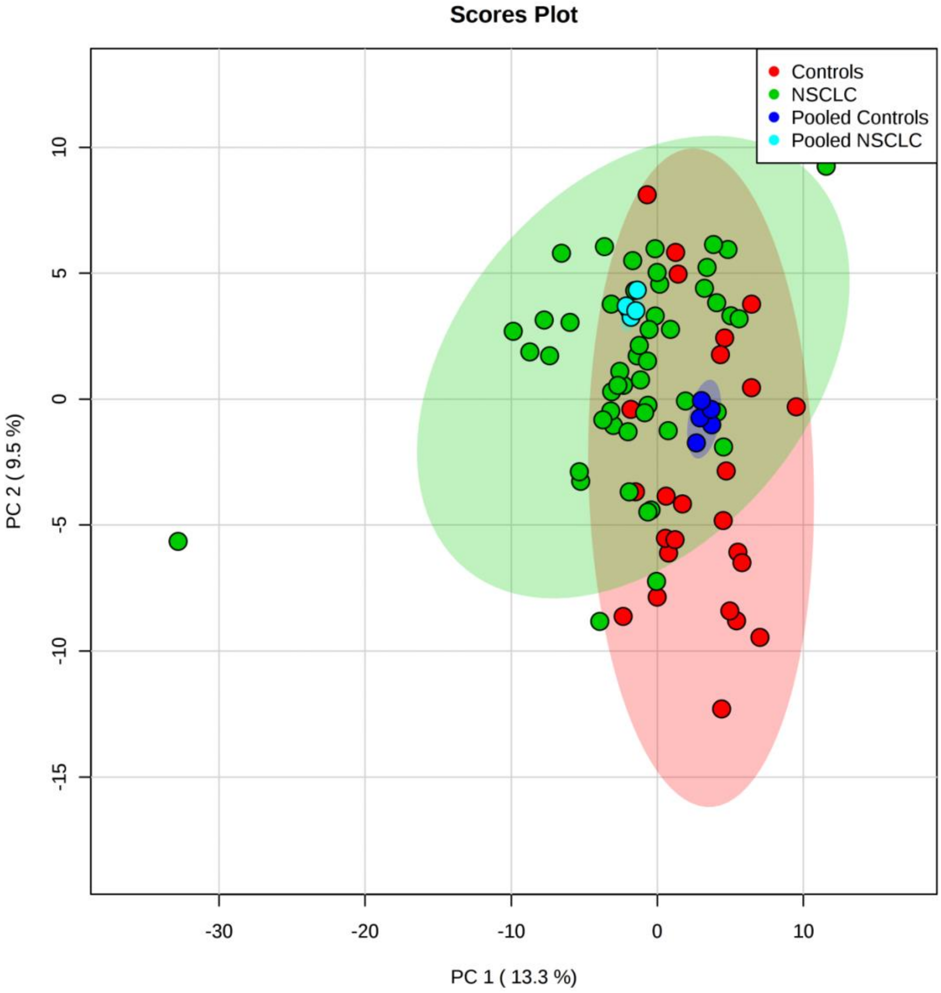

Fig. S1 Scores plot between the first and the second principal components (with the explained variances shown in brackets) obtained in PCA of global metabolite profiles in serum in two studied groups: NSCLC (non-small cell lung cancer, n = 50, green dots) and controls (n = 25, red dots), as well as in pooled NSCLC (n = 5, light blue dots) and pooled controls (n = 5, dark blue dots)
